# Influence of beamlet width on dynamic IMRT plan quality in nasopharyngeal carcinoma

**DOI:** 10.7717/peerj.13748

**Published:** 2022-08-05

**Authors:** Manya Wu, Jinhui Jin, Zhenghuan Li, Fantu Kong, Yadi He, Lijiang Liu, Wei Yang, Xiangying Xu

**Affiliations:** 1 Department of Radiation Oncology, The Third Affiliated Hospital of Sun Yat-Sen University, Guangzhou, Guangdong, China; 2 School of Biomedical Engineering, Southern Medical University, Guangzhou, Guangdong, China

**Keywords:** IMRT, Beamlet width, Plan quality, Delivery efficiency, NPC

## Abstract

**Objective:**

This study aimed to identify the effects of beamlet width on dynamic intensity-modulated radiation therapy (IMRT) for nasopharyngeal carcinoma (NPC) and determine the optimal parameters for the most effective radiotherapy plan.

**Methods:**

This study evaluated 20 patients with NPC were selected for dynamic IMRT. Only the beamlet width in the optimization parameters was changed (set to 2, 4, 6, 8, and 10 mm that were named BL02, BL04, BL06, BL08, and BL10, respectively) to optimize the results of the five groups of plans. Using the plan quality scoring system, the dose results of the planning target volumes (PTVs) and organs at risks (OARs) were analyzed objectively and comprehensively. The lower the quality score, the better the quality of the plan. The efficiency and accuracy of plan execution were evaluated using monitor units (MUs) and plan delivery time (PDT).

**Results:**

The BL04 mm group had the lowest quality score for the targets and OARs (0.087), while the BL10 mm group had the highest total score (1.249). The BL04 mm group had the highest MUs (837 MUs) and longest PDT (358 s). However, the MUs range of each group plan was below 100 MUs, and the PDT range was within 30 s. In the BL02, BL04, BL06, BL08, and BL10 plans, <5 MUs segments accounted for 33%, 16%, 24%, 33%, and 40% of total segments, respectively, with which the lowest was in the BL04 mm group.

**Conclusion:**

Smaller beamlet widths have not only reduced OARs dose while maintaining high dose coverage to the PTVs, but also lead to more MUs that would produce greater PDT. Considering the quality and efficiency of dynamic IMRT, the beamlet width value of the Monaco treatment planning system set to 4 mm would be optimal for NPC.

## Introduction

Intensity-modulated radiation therapy (IMRT) has been considered as a common treatment for a variety of cancers, because it improves the dose coverage of tumors and reduces the dose exposure in normal tissues (Verbakel et al., 2009; Tham et al., 2009; Doornaert et al., 2011). Over the last decade, IMRT has been a major field of research in the treatment of nasopharyngeal carcinoma (NPC) owing to its dosimetric advantages (Guo et al., 2021; Lee et al., 2012; Sayah et al., 2020). In automatic IMRT optimization, the quality and efficiency of plan are closely associated with a number of different parameters (Pakela et al., 2020; Burmeister et al., 2004; Chen et al., 2020; Wang et al., 2018). The general approach for this technique is to broke up each beam into several beamlets, which are small units that can be modulated with the aid of a computer program. Generally, IMRT planning is implemented in two steps: first, the ideal fluence map is optimized; second, the ideal fluence is converted into an executable form after considering the shape and physical limitations of the multi-leaf collimator (MLC) sequence (Webb, 2003). The beamlet width parameter takes an important role in the fluence distribution when generating an ideal fluence map in the first step (Zhang et al., 2020). The effects of the beamlet width on IMRT plans have been studied by several authors (Nill et al., 2005). Among their research, the beamlet widths were generally chosen the range of 0.1 cm to 1.0 cm using the treatment planning system (TPS) (Zhang et al., 2005; Kim et al., 2011). However, there have been no reports regarding beamlet width optimization in terms of IMRT plan quality and efficiency in NPC. Therefore, the purpose of this study was to explore the influence of the beamlet width parameter on the quality and efficiency of IMRT plans for NPC to provide a useful reference for clinical treatment planning.

## Materials & Methods

### Study design and patients

The retrospective study was approved by the medical ethics committee of the Third Affiliated Hospital of Sun Yat-sen University (Approval No. [2022]-02-043-01). The need for written informed consent was waived owing to the retrospective nature of the study.

A total of 20 patients with NPC aged 25–60 years and who received IMRT in the Department of Oncology Radiotherapy between February and April 2020 were evaluated. Among them, 16 and four patients were male and female, respectively.

### Simulation and contouring

**Figure 1:**
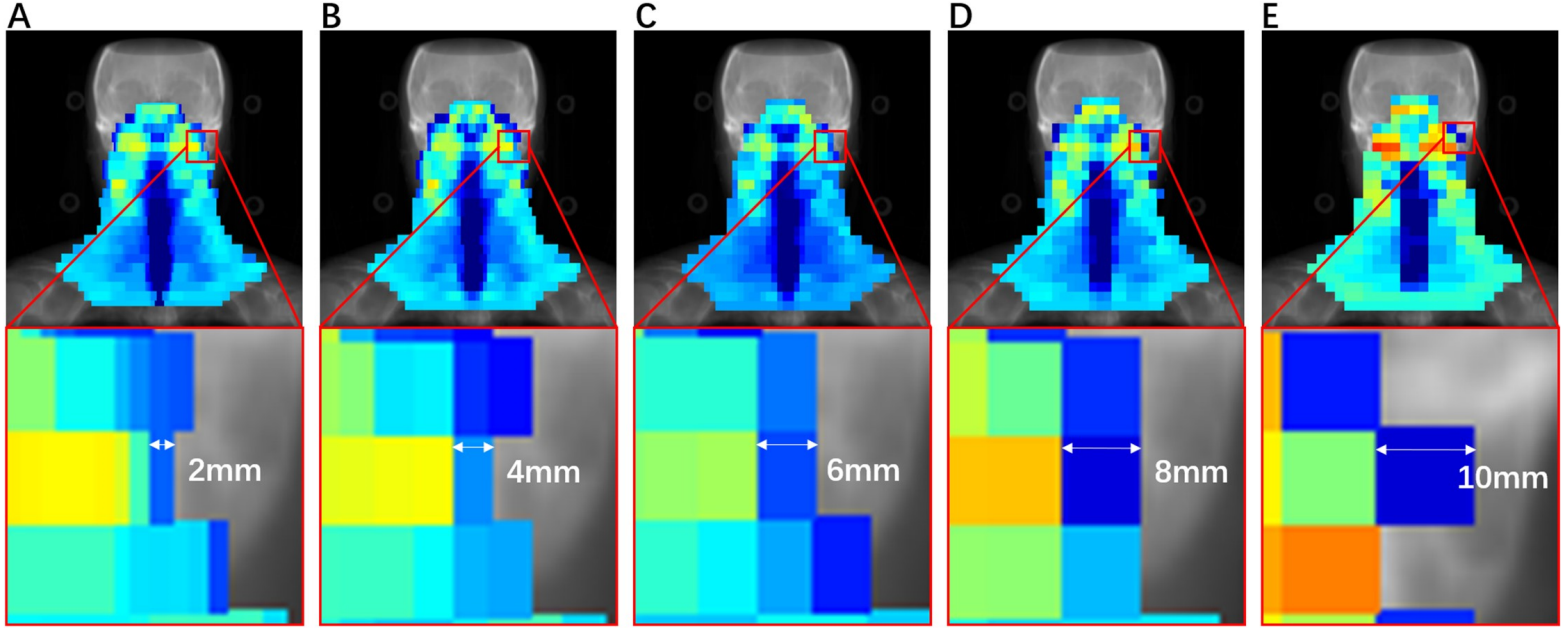
Influence segmentation map with beamlet width of (A) 2, (B) 4, (C) 6, (D) 8, and (E) 10 mm.

The Elekta synergy accelerator used for irradiation was originally equipped with an MLCi2 offering 40 pairs of leaves with one cm width. Figure 1 shows the division of the fluence map with different beamlet widths. All patients were fixed with head, neck, and shoulder stabilizers and polyurethane foam sealing agent in the supine position. Siemens computed tomography was used, and the scanning range was from the top of the head to two cm below the clavicle head. The reconstructed images were transmitted to the Monaco TPS. Following the International Commission on Radiation Units and Measurements (ICRU) report number 62, the gross target volume (GTV) was divided into the primary tumor gross target volume and neck metastatic lymph node gross target volume. The clinical target volume was divided into high-risk clinical target volume (CTV) and low-risk CTV, which are mainly used to prevent exposure. The PTV was defined as a uniform three-dimensional expansion of the CTV or GTV by three mm. The OARs included the lens, optic nerves, optic chiasm, eyes, spinal cord, brainstem, pituitary, parotids, temporal lobes, mandibular joints, and mandibles.

### Planning

All plans used X-rays with an energy of 6 MV in the Monaco TPS for dynamic IMRT planning, which were set to nine beams at 160°, 120°, 80°, 40°, 0°, 320°, 280°, 240°, and 200°. The PTVs and OARs adopted naming rules as recommended by the American Association of Physicists in Medicine task group-263 report (Mayo et al., 2015). The prescribed doses of the primary tumor (PTVnx), neck metastatic lymph nodes (PTVnd), the high-risk clinical target region (PTV1), and the low-risk clinical target region (PTV2) were 70, 66, 64, and 56 Gy, respectively. The prescribed dose was required to contain at least 95% of the target volumes and the OARs, as shown in Table 1. The beamlet widths were set to 2, 4, 6, 8, and 10 mm, and the plans were named BL02, BL04, BL06, BL08, and BL10, respectively. The other parameters and constraint functions remained unchanged. To ensure the accuracy of the calculation, a 2-mm calculation grid was used, and the plans met the prescribed doses and limits of each OAR (Mnejja et al., 2020).

**Table 1:** Dose constraints to the OARs of the IMRT plans with five different beamlets for 20 NPC patients.

OARs	Parameter	Constraint
Lens	D_2%_(Gy)	≤8
Eyes	D_mean_ (Gy)	≤5
Parotids	V_30_Gy (%)	≤5
Mandibular joints	D_2%_ (Gy)	≤70
Pituitary	D_2%_ (Gy)	≤60
Mandibles	D_2%_ (Gy)	≤70
Brainstem	D_2%_ (Gy)	≤54
Spinal cord	D_2%_ (Gy)	≤45
Optic nerves	D_2%_ (Gy)	≤54
Optic chiasm	D_2%_ (Gy)	≤54
Temporal lobes	D_2%_ (Gy)	≤65

### Plan evaluation

The quality score was used to compare the quality of the plans (Bohsung et al., 2005). However, the plan results obtained from some optimized conditions demonstrated that the protective effect of OARs was good, but the dose distribution of the tumors was poor. In such cases, it was difficult to evaluate the overall quality of the plan. To overcome this problem, we used a quality score system to analyze different plans. The plan quality score was used to compare the overall accomplishments of the same dose from the different plans. The group with the best results among the five groups was used as the reference group. A higher plan quality score indicated a less effective overall quality of the plan. A total score of 0 indicated that all indicators of the plan were better than those of the other plans. For example, the minimum dose of the brainstem in the BL10 mm group was 50.90 Gy, with the lowest being 49.10 Gy in the BL04 mm group. Using BL04 mm as the reference group, according to formulas (1) and (2), the score of the brainstems was 0.037 in the BL10 mm group and 0.000 in the BL04 mm group. Other targets involved in the evaluation were also evaluated using the same formula. The overall quality score of the plan was obtained by adding the scores for all the items.

(1) \begin{eqnarray*}{S}_{j}= \left\vert \frac{{M}_{j}-{\mathrm{C}}_{j}}{{C}_{j}} \times {P}_{j} \right\vert \end{eqnarray*}

(2) \begin{eqnarray*}{S}_{D}=\sum _{j=1}^{k}{S}_{j}\end{eqnarray*}

where *M*_*j*_ is the actual dose to the j-th anatomical structure (PTV or OAR); *C*_*j*_ is the target dose; *P*_*j*_ is the weighting factor of the anatomical structure (1 in this study); *S*_*D*_ is the overall quality score of the plan; *k* is the number of targets included in the evaluation; and *S*_*j*_ is the *j*-th sub-item quality score.

### Dose-volume histogram evaluation

For the target area, the evaluation parameters were target coverage (TC), homogeneity index (HI), conformity index (CI), and D_2%_. Eq. (3) is the calculation formula for TC. HI and CI were calculated following the ICRU83 report using formulas Eqs. (4) and (5), respectively,

(3) \begin{eqnarray*}\mathrm{TC}(\text{%})=(\mathrm{T}{\mathrm{V }}_{\mathrm{PI}}/\mathrm{TV })\times 100\text{%}\end{eqnarray*}

(4) \begin{eqnarray*}\mathrm{HI}=({D}_{2\text{%}}/{\mathrm{D}}_{98\text{%}})/{\mathrm{D}}_{50\text{%}}\end{eqnarray*}

(5) \begin{eqnarray*}\mathrm{CI}={ \left( \mathrm{T}{\mathrm{V }}_{\mathrm{PI}} \right) }^{2}/(\mathrm{TV }\times {\mathrm{V }}_{\mathrm{ PI}})\end{eqnarray*}

where D_2%_ is the dose containing 2% of the target volume, D_98%_ is the dose containing 98% of the target volume, and D_50%_ is the dose containing 50% of the target volume. The smaller the HI value, the more uniform the target dose. TV is the PTV, TV_PI_ is the volume of the target area included in the prescribed dose, and V_PI_ is the total volume of the prescribed dose line. The closer the CI value is to 1, the better the conformity of the target area.

The MUs of segment is an important parameter in IMRT planning as it can directly affect the plan’s quality and efficiency. Therefore, the influence of the MUs should be considered in plan optimization. When the minimum MUs of a segment is increased from four to eight, the number of segments is reduced by 36.5%; however, the dose of the PTVs and OARs did not change significantly. Therefore, the minimum MUs should be set to 5, considering the quality of the plan.

### Statistical analysis

The results were presented as the mean ± standard deviation. The plan results of the optimal group were evaluated using the quality score system to obtain better dose parameters than those obtained using other alternative beam widths. The *t*-test for normally distributed data and the rank-sum test for non-normally distributed data, followed by Bonferroni’s correction, were used to the intergroup comparison for dosimetric parameters and measurement results. SPSS version 22.0 (IBM Corp., Armonk, NY, USA) was used for all statistical analyses, and *p* < 0.05 was considered statistically significant.

## Results

### Overall quality score of plans

**Figure 2:**
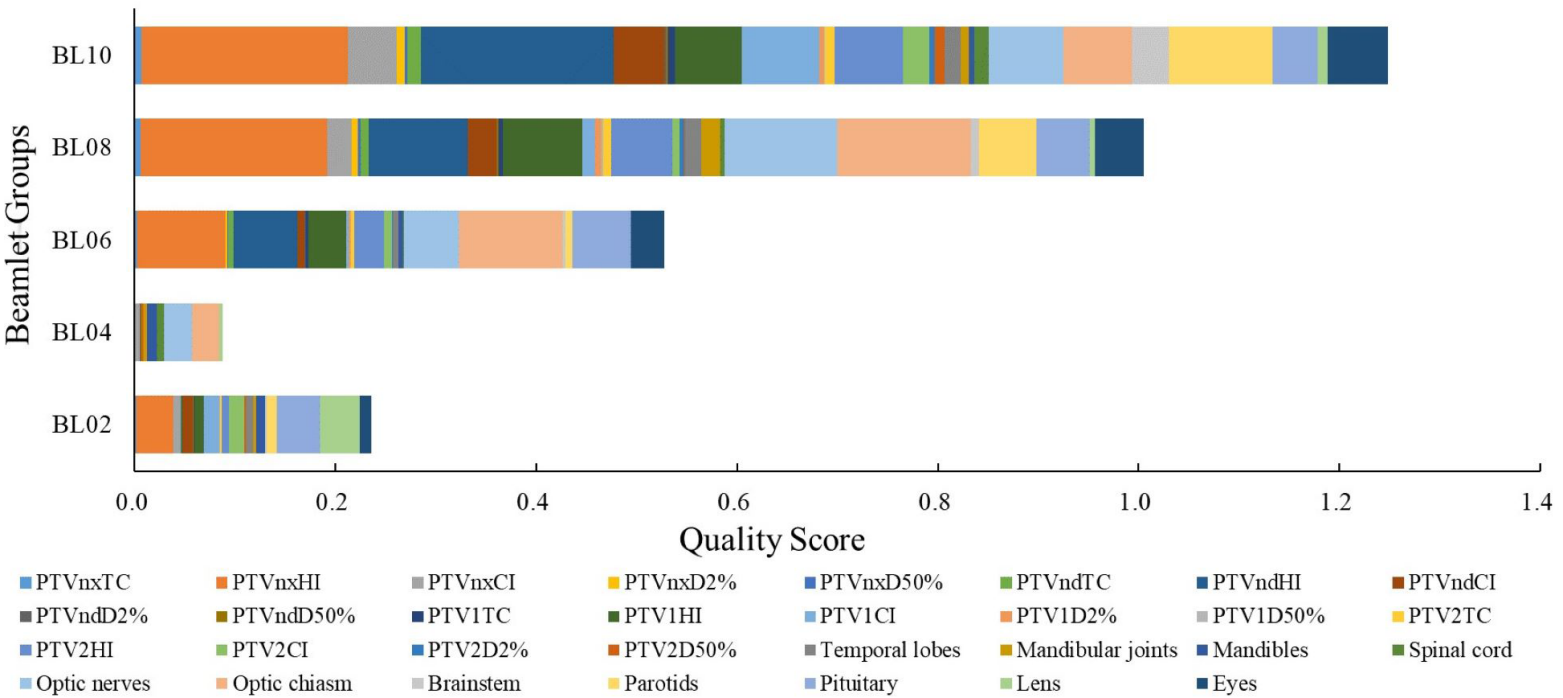
Overall score of dynamic IMRT plan for different beamlet widths.

The TC, HI, D_2%_, D_50%_, PTV, and Dmax of the OARs were scored according to the planning scoring system, and the highest plan quality was determined according to the score. A lower score indicated a higher overall plan quality. The results for the overall quality scores of the dynamic IMRT plans with different beamlet widths are presented in Fig. 2. The overall PTVs and OARs scores of the BL02, BL04, BL06, BL08, and BL10 mm groups were 0.236, 0.087, 0.528, 0.1, and 1.249, respectively. The BL04 group had the lowest score (0.087), indicating that the overall quality of the plan was the most effective. For the TC of PTVs, the five groups scored 0.111, 0.009, 0.257, 0.548, and 0.807. The BL04 mm group had the lowest total target area score of 0.009, indicating that it had the highest target coverage. For OARs, the five groups scored 0.125, 0.079, 0.270, 0.457, and 0.441, and the BL04 mm group had the lowest total score, indicating that it had the lowest radiation dose.

### PTV

Based on the results of the overall quality scoring system, the BL04 mm group had the lowest total score, indicating that it had the most effective overall quality of the plan. As such, the BL04 mm group was set as the reference group. Table 2 provides the PTVs of the CI, HI, TC, and D_2%_ for the BL02, BL04, BL06, BL08, and BL10 mm groups. Although the CI, HI and D_2%_ values were no significant differences among all five groups, the TC was highest with the plan using beamlet widths of four mm. Moreover, compared to the plan with beamlet widths of four mm, the *p* value of PTVnd for beamlet widths of 6, 8 and 10 mm reached statistical significance (*p* = 0.015, *p* = 0.014, and *p* = 0.042, respectively).

**Table 2:** PTVs of CI, HI, TC and D_2%_ for plans with five different Beamlets.

		BL02	BL04	BL06	BL08	BL10	*p*1	*p*2	*p*3	*p*4
CI	PTVnx	0.62 ± 0.05	0.62 ± 0.04	0.62 ± 0.04	0.61 ± 0.05	0.59 ± 0.05	0.541	0.873	0.562	0.478
	PTVnd	0.14 ± 0.07	0.15 ± 0.08	0.15 ± 0.08	0.14 ± 0.07	0.14 ± 0.07	0.908	0.943	0.878	0.811
	PTV1	0.47 ± 0.08	0.48 ± 0.08	0.48 ± 0.08	0.47 ± 0.07	0.47 ± 0.08	1.000	0.939	0.619	0.762
	PTV2	0.76 ± 0.05	0.77 ± 0.05	0.77 ± 0.05	0.75 ± 0.05	0.76 ± 0.05	0.824	0.958	0.892	0.745
HI	PTVnx	0.07 ± 0.02	0.07 ± 0.02	0.07 ± 0.02	0.08 ± 0.02	0.08 ± 0.02	0.394	0.567	0.365	0.424
	PTVnd	0.07 ± 0.01	0.07 ± 0.01	0.08 ± 0.01	0.08 ± 0.01	0.09 ± 0.01	0.958	0.128	0.394	0.917
	PTV1	0.15 ± 0.02	0.14 ± 0.01	0.15 ± 0.01	0.16 ± 0.02	0.15 ± 0.03	0.535	0.811	0.464	0.447
	PTV2	0.27 ± 0.02	0.27 ± 0.02	0.28 ± 0.02	0.29 ± 0.02	0.29 ± 0.03	0.875	0.574	0.644	0.960
TC	PTVnx	98.04 ± 1.2	98.16 ± 1.10	97.92 ± 1.16	97.60 ± 1.09	97.50 ± 1.10	0.678	0.770	1.000	0.895
	PTVnd	98.25 ± 1.16	98.31 ± 1.23	97.82 ± 0.90	97.49 ± 0.86	96.96 ± 0.93	0.436	0.015	0.014	0.042^a^
	PTV1	98.23 ± 0.84	98.30 ± 0.80	98.00 ± 0.71	97.84 ± 0.61	97.59 ± 0.61	0.740	0.535	0.436	0.403
	PTV2	98.01 ± 1.05	98.15 ± 1.01	97.76 ± 0.92	97.40 ± 0.75	97.16 ± 0.42	0.497	0.719	0.397	0.018
D_2%_	PTVnx	74.56 ± 0.33	74.55 ± 0.27	74.72 ± 0.32	75.05 ± 0.54	75.15 ± 0.58	0.826	0.556	0.193	0.132
	PTVnd	70.96 ± 0.30	71.01 ± 0.28	71.00 ± 0.26	71.01 ± 0.22	71.09 ± 0.20	0.682	0.93	0.755	0.476
	PTV1	74.26 ± 0.34	74.27 ± 0.30	74.40 ± 0.35	74.70 ± 0.56	74.59 ± 1.05	0.746	0.561	0.132	0.065
	PTV2	73.48 ± 0.43	73.49 ± 0.40	73.56 ± 0.44	73.79 ± 0.57	73.89 ± 0.64	0.890	0.686	0.243	0.082

**Notes.**

*p*1, *p*-value of comparison between BL02 and BL04; *p*2, *p*-value of comparison between BL06 and BL04; *p*3, *p*-value of comparison between BL08 and BL04; *p*4, *p*-value of comparison between BL10 and BL04.

a Statistically significant according to Bonferroni correction.

### OAR

Table 3 provides the doses required in the OARs of IMRT plans with different beamlet widths for the 20 patients with NPC. The D_2%_ of the temporal lobes, mandibular joints, brainstem, parotids, pituitary, and eyes were the lowest in the BL04 mm group. The total score for BL04 mm was the lowest, indicating that the BL04 mm group plan had the most effective overall quality. Thus, the BL04 mm group was set as the reference group. The results of the BL08 mm and BL10 mm group changed slightly in the temporal lobes (*p* = 0.013, *p* = 0.015), parotids (both *p* = 0.001), and eyes (*p* = 0.016, *p* = 0.024) as evidenced by statistically significant differences, compared to the BL04 mm group, respectively. Though the scores of the optic nerves were significant different (*p* = 0.027) between BL08 mm and BL04 groups, there were no significant differences between other three group plans. Both compared with BL04 mm group respectively, doses to the optic chiasm between the BL06 mm and BL08 mm groups significantly increased (*p* = 0.021, *p* = 0.004), while the plan with beamlet widths of two mm and 10 mm did not reach statistical significance.

**Table 3:** Dose to OARs of IMRT plans with five different Beamlets for (*n* = 20 patients).

OARs	Parameter	BL02	BL04	BL06	BL08	BL10	*p*1	*p*2	*p*3	*p*4
Temporal lobes	D_max_(Gy)	72.39 ± 3.63	71.91 ± 4.03	72.28 ± 3.84	73.03 ± 4.03	73.03 ± 4.03	0.237	0.315	0.013	0.015
Mandibular Joints	D_max_(Gy)	66.29 ± 5.30	66.27 ± 5.56	66.03 ± 5.89	67.29 ± 5.68	66.56 ± 5.18	0.818	0.476	0.286	0.433
Mandibles	D_max_(Gy)	68.02 ± 3.97	68.06 ± 4.13	67.74 ± 4.38	67.40 ± 3.70	67.77 ± 3.95	0.833	0.247	0.202	0.387
Spinal cord	D_max_(Gy)	42.87 ± 1.33	43.18 ± 3.06	42.89 ± 1.71	43.07 ± 1.33	43.49 ± 1.23	0.614	0.633	0.843	0.661
Optic nerves	D_max_(Gy)	40.25 ± 16.24	41.35 ± 16.34	42.48 ± 15.5	44.77 ± 14.38	43.28 ± 13.75	0.418	0.639	0.027	0.306
Optic chiasm	D_max_(Gy)	38.03 ± 17.16	39.06 ± 17.30	41.96 ± 14.46	43.08 ± 14.81	40.61 ± 13.38	0.127	0.021	0.004	0.385
Brainstem	D_max_(Gy)	49.18 ± 2.25	49.10 ± 2.22	49.19 ± 2.48	49.50 ± 2.51	50.90 ± 4.65	0.600	0.704	0.239	0.074
Parotids	V_30_(%)	49.01 ± 6.73	48.51 ± 5.54	48.86 ± 6.17	51.3 ± 7.68	53.53 ± 10.11	0.364	0.412	0.001	0.001^*^
Pituitary	D_max_(Gy)	55.16 ± 11.25	52.88 ± 11.54	55.97 ± 8.60	55.68 ± 9.73	55.26 ± 9.53	0.229	0.114	0.163	0.224
Lens	D_max_(Gy)	7.53 ± 2.33	7.27 ± 2.19	7.24 ± 2.14	7.28 ± 2.16	7.31 ± 2.24	0.137	0.926	0.961	0.894
Eyes	D_mean_(Gy)	7.61 ± 2.39	7.52 ± 2.37	7.78 ± 2.28	7.88 ± 2.32	7.98 ± 2.29	0.478	0.087	0.016	0.024

**Notes.**

D_max_; the dose containing 2% of the target volume (D2%); *p*1, *p*-value of comparison between BL02 and BL04; *p*2, *p*-value of comparison between BL06 and BL04; *p*3, *p*-value of comparison between BL08 and BL04; *p*4, *p*-value of comparison between BL10 and BL04.

* Statistically significant according to the Fisher least significant difference (LSD) correction.

### Delivery efficiency

**Figure 3:**
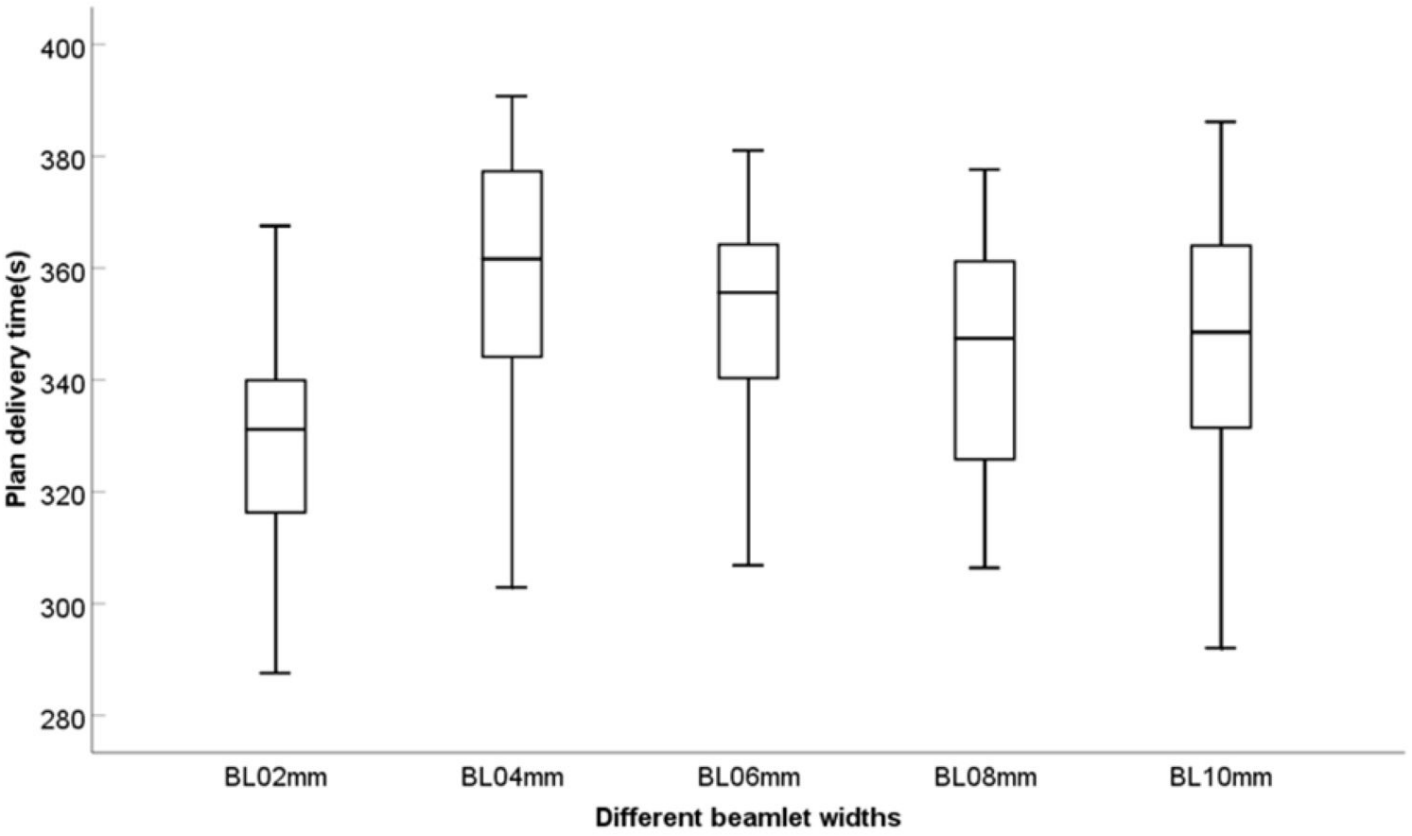
Delivery efficiency of the five different beamlet widths.

The MUs of the BL04 mm group was 837, which was higher than that of the other groups. The BL04 mm group had the longest plan delivery time (PDT) of 358 s. However, the difference was not significant, and the range was within 30 s. The PDT tended to shorten with an increase in the beamlet width, as shown in Fig. 3. Furthermore, the ratio of the number of segments <5 MUs to the total number of segments was calculated. Table 4 shows the results of MUs, PDT, and the ratio of the number of segments <5 MUs for the five IMRT plans. Therefore, the BL04 mm group had the higher MUs than the other groups.

## Discussion

The study found that beamlet width not only affects the dose distribution of the PTVs and OARs, but also changes the MU and PDT. When designing an IMRT plan for NPC, the dosimetry effects, segments, and plan execution efficiency should be evaluated because of the different settings of the optimized parameters. Although the beamlet width is a crucial parameter in IMRT optimization, it is also easily overlooked. The beamlet width directly affects the fluence segmentation in the first step of the intensity modulation optimization and indirectly affects the segment optimization in the second step. Theoretically, the smaller is the combined value, the finer is the fluence map, which may lead to a more effective dose distribution (Nguyen et al., 2015). However, this may result in a smaller segment (Lam et al., 2019). Furthermore, the smaller the segment, the lower the treatment efficiency (Mohan et al., 2000). Only segments less than five MUs are counted. Because the TPS cannot directly read the area of the segment, the area of the segment is not statistically analyzed; thus, the beamlet width may affect more small-area segments. Therefore, the most effective plan quality and treatment efficiency could be obtained by comparing different beamlet widths (2, 4, 6, 8, and 10 mm).

A comparison of the results of the five groups of different beamlet widths showed that the BL04 mm group had the lowest score. Thus, the BL04 mm group was set as the reference group. The TC of the five groups was above 97% and that of the BL04 mm group was higher than those of the other groups. HI and D_2%_, which represents the highest dose of the PTV, decreased as the beam width decreased. This indicates that the target dose distribution was more uniform and did not lead to a reduction in the CI. The higher the conformity, the closer is the CI (between 0 and 1). Therefore, a reduction in beamlet width can improve the quality of the target area. For the plan with beamlet widths of eight mm, the exposure dose of the OARs did not significantly change; however, most OARs had the lowest exposure dose in the BL04 mm group. A comprehensive evaluation of the overall score of the target area and OARs revealed that the BL04 group plan had the lowest score (0.087), indicating that it had the most effective overall target dose distribution and PTV protection. The BL10 mm group had the highest score (1.249), indicating that it had the least effective overall target dose distribution and protection of the OARs.

**Table 4:** MUs and PDT of IMRT plans.

Parameter	BL02	BL04	BL06	BL08	BL10
MUs	739.80 ± 43.74	843.03 ± 52.62	776.97 ± 30.51	728.17 ± 42.41	701.71 ± 30.67
5MUs/plan MUs (%)	32.35 ± 6.27	15.70 ± 7.67	24.10 ± 6.41	32.75 ± 6.32	39.50 ± 5.89
PDT(s)	328.29 ± 20.67	358.92 ± 23.55	351.27 ± 18.37	344.85 ± 22.12	347.65 ± 25.16

Compared with the other groups, the BL04 mm group had relatively lower execution efficiency and relatively higher MUs. However, the range of MUs for each group was less than 100 MUs, and the MUs gradually decreased for the plan with beamlet widths of six mm. Although the BL04 mm group had the longest PDT on average, it was only 30 s longer than those with the shortest PDT, which is not significant in clinical trials. Different beamlet widths result in different numbers of MUs and PDT. The larger the number of MUs, the longer the PDT, increasing the probability of patient displacement and the target dose in a single treatment (Younge et al., 2012; Jiang et al., 2005). In this study, the MUs of the BL04 mm group was 837, which was higher than that of the other groups. The BL04 mm group had the longest PDT of 358 s. However, the difference was not significant, and the range was within 30 s. Among all plan groups, the BL04 mm group has the lowest ratio of the number of sub-wilds <5 MUs to the total number of subfields (15.70%); the proportion was also the highest in BL10 (39.50%). The PDT tended to shorten with an increase in the beamlet width, which determines the fluence map. However, the final segment is determined based on the physical limit of the MLC (Jin et al., 2010). Therefore, the dose distribution is not affected because the beamlet width continues to decrease. To meet the dose requirements of PTVs and OARs, the dose distribution should not change considerably with an increase in the beamlet width.

The dose-volume histogram, which reflects the quantitative relationship between the dose and volume, can be used as reference in radiotherapy planning (Nguyen et al., 2015). However, information on the spatial distribution of the doses is lacking. The MUs and extension of PDT negatively affect the treatment efficiency. Furthermore, the evaluation plan needs to comprehensively consider the significant relationship between the dose distribution and efficiency. If the quality and efficiency cannot be considered concurrently, the quality of the plan should be the main focus. In this study, although the BL04 mm group showed a slightly lower execution efficiency, the plan quality was the highest. In addition, although the proportion of small MUs count segments was the lowest, the plan of the BL04 mm group was still considered the most beneficial for patients.

In this study, we investigated 20 patients with NPC who underwent IMRT with an MLC width of one cm as a preliminary attempt. Accordingly, this study has some limitations, including the relatively small sample size. Future studies need to replicate and extend these results by including larger samples and more types of cancer in which beamlet width influences intensity modulation (*e.g.*, cervical cancer and lung cancer). Additionally, the influence of beamlet width on the treatment plans of different linear accelerator models should be further investigated.

## Conclusions

In generally, IMRT plans of NPC that are generated with smaller beamlet widths have not only reduced OARs dose while maintaining high dose coverage to the PTVs, but also lead to more MUs that would produce greater PDT. According to our findings, IMRT plans with beamlet widths of four mm show a clear benefit in terms of a trade-off between plan quality and efficiency for NPC, and can optimally satisfy the clinical requirements.

## Supplemental Information

10.7717/peerj.13748/supp-1 Supplemental Information 1 The values of CI, HI, TC, and D_2% for the target area

10.7717/peerj.13748/supp-2 Supplemental Information 2 Abbreviations

## Additional Information and Declarations

### Data Availability

The following information was supplied regarding data availability: The raw measurements are available in the Supplementary Files.
