# Supplementary material for: Influence of beamlet width on dynamic IMRT plan quality in nasopharyngeal carcinoma
Source: PeerJ. 2022 Aug 5;10:e13748. doi: 10.7717/peerj.13748 (PMC9359131; doi:10.7717/peerj.13748)
Supplement: Supplemental Information 2 [file peerj-10-13748-s002.docx]

Abbreviations: IMRT = intensity-modulated radiation therapy, NPC = nasopharyngeal carcinoma, CTV = clinical target volume, DVH = dose-volume histogram, MLC = multi-leaf collimator, GTV = gross tumor volume, Gy = Gray (unit), ICRU = International Commission of Radiation Units, TPS = treatment planning system, MU = monitor unit, OAR = organs at risk, PTV = planning target volume, TC = target coverage, HI = homogeneity index, CI = conformity index, PDT = plan delivery time.
